# Prokaryotic Expression and Binding Characteristics of Odor-Binding Protein GqinOBP10 in *Gynaephora qinghaiensis*

**DOI:** 10.3390/ijms262110502

**Published:** 2025-10-29

**Authors:** Zhanling Liu, Dejing Tang, Youpeng Lai, Shujing Gao, Haibin Han, Yuantao Zhou

**Affiliations:** 1 College of Agriculture and Animal Husbandry, Qinghai University, Xining 810016, China; liu20000607ling@163.com (Z.L.); 15319205805@163.com (D.T.); 2 Institute of Plant Protection, Qinghai Academy of Agriculture and Forestry, Xining 810016, China; 3 Institute of Grassland, Chinese Academy of Agricultural Sciences, Hohhot 010010, China; gaoshujing@caas.cn (S.G.); hhb.25@163.com (H.H.)

**Keywords:** *Gynaephora qinghaiensis*, GqinOBP10, prokaryotic expression, fluorescent competitive binding, molecular docking, RNAi

## Abstract

*Gynaephora qinghaiensis* is a major grassland pest common in the alpine meadows of the western plateau of China, and its biological behavior is affected by the synergy of a variety of chemicals in the environment. OBPs can dissolve and transport odor molecules such as volatile plant compounds through lymphatic fluid, which plays an important olfactory-to-olfactory role. However, the specific function of OBPs in the interaction mechanism between moths and volatile plant compounds is still unknown. The purpose of this study was to analyze the binding characteristics of GqinOBP10 and its volatile plant compounds in moths and to explore its role in the olfactory perception mechanism of moths so as to study the corresponding target ligands and achieve green control. The purified GqinOBP10 was subjected to fluorescence competitive binding to eight ligands. The 3D modeling of GqinOBP10 was carried out by the SWISS-MODEL website, and the molecular docking was carried out by Autodock 4.2.6 software, and the binding of GqinOBP10 to eight ligands was simulated and verified. The results showed that the cloned strain with the full length of GqinOBP10 was cloned. The fluorescence competition binding results showed that GqinOBP10 had strong binding ability to eight volatile plant compounds, among which the binding ability to 2-Amino-1-phenylethanol and 2-Oleoylglycerol was the strongest, and had high binding ability with the other six ligands. The molecular docking results showed that the binding energy of GqinOBP10 and eight odorant molecules was negative, and all of them could form 1~4 hydrogen bond for binding, among which the binding performance with 2-Oleoylglycerol was the best. The findings suggest that *dsOBP10* injection leads to a notable decrease in both the expression levels of *GqinOBP10* and the antennal potential response in male and female tissues. This indicates that GqinOBP10 is likely crucial for the localization and recognition of host plants in *G. qinghaiensis*. By silencing *GqinOBP10*, the olfactory perception of host volatiles is significantly impaired, highlighting the protein’s importance in the caterpillars’ ability to detect and respond to their environment. These insights provide a valuable basis for developing targeted attractants, potentially enhancing pest management strategies by manipulating olfactory cues in these caterpillars. Further research could explore the specific mechanisms by which GqinOBP10 influences olfactory perception and host plant selection.

## 1. Introduction

In recent years, the problem of grassland desertification has received extensive attention. As an important pest of plateau meadows, *G. qinghaiensis* (Lepidoptera, Lymantriidae) occurs prevailingly in the Qinghai–Tibet Plateau. When the damage appears, the population density can reach 1000 heads per square meter, and the grass is eaten as soon as it turns green, which accelerates the degradation of grassland and seriously hinders the development of the local animal husbandry economy [1]. To date, a total of 15 species of the Gynaephora have been found, 8 of which are endemic to the Tibetan Plateau [2,3] and are distributed in Qinghai, Gansu, Sichuan, Tibet, and other regions. Most of its volatile plants are Poaceae, Cyperaceae, Leguminosae, Polygonaceae, Rosaceae, and other high-quality grasses, especially the *Festuca rubra* (Poaceae, Festuca). Several studies have indicated that *G. qinghaiensis* exhibits remarkable adaptability to extreme environmental conditions, including intense ultraviolet radiation, hypoxia, and cold temperatures. Additionally, it possesses a strong reproductive capacity. The larvae are equipped with poison glands on their backs, which can trigger allergic reactions in both domestic animals and humans; consequently, effective prevention and control measures pose significant challenges [4,5]. Since the 1960s, significant manpower and material resources have been dedicated to pest control efforts. Nevertheless, as of now, the application of chemical agents remains the most effective and practical method for managing *G. qinghaiensis*. However, excessive pesticide use has led to increased resistance among pests, while pesticide residues and environmental pollution pose serious challenges to the sustainable development of grassland ecosystems [6]. Therefore, it is imperative to explore more efficient, environmentally friendly, and sustainable prevention and control strategies.

Olfactory manipulation represents an innovative form of green control technology, demonstrating significant potential for practical applications [7]. The olfactory system of insects plays an important role in their olfactory recognition. In the context of interspecific communication between herbivorous insects and plants, insects utilize their highly sensitive and specific olfactory systems to perceive, detect, distinguish, and interpret critical information conveyed by volatile semiochemical substances in their environment. This capability drives essential behaviors such as locating volatile plants, feeding strategies, evading natural predators, mating rituals, and selecting oviposition sites [8,9,10,11]. Odor-binding proteins (OBPs) of Lepidoptera, as key water-soluble carrier proteins that distinguish various odor molecules, play an important role in guiding olfactory-related behaviors. According to previous studies, they can be divided into general odorant-binding proteins (GOBPs), antenna special proteins (ASPs), and pheromone binding proteins (PBPs) [12,13]. OBPs are rich water-soluble acidic proteins with six conserved cysteine residues, which form three linked disulfide bonds in pairs. A stable and compact three-dimensional hydrophobic binding cavity is generated to bind and protect small hydrophobic ligands [14,15,16]. Studies have shown that OBPs are also involved in insect regeneration and development as carriers of visual pigments and are associated with insecticide resistance [17]. Volatile plants, food, insects of the opposite sex, habitats, and natural enemies all have their own specific chemical signals that form a communication network between insects and their environment (insect odorscapes). Odor molecules in the environment include compounds such as volatile organic compounds (VOCs), volatile plant compounds (VPCs), and sex pheromones. External fat-soluble micromolecular compounds perceived by insects can only be recognized when combined with OBPs [10,18,19,20]. OBPs selectively bind to external odor molecules, enter lymphatic fluid through the epidermal pores of sensilla trichodea or sensilla basiconica on antennae, bind to lymphocytes and transfer to odorant receptors (ORs) on the dendritic membrane of olfactory receptor neurons, activate the olfactory signal transduction pathway, and thus control insects to make corresponding behavioral responses [21,22]. Thus, interference with the corresponding odor-binding protein gene can interrupt the chemical communication between species and between pests and their host plants, achieving the purpose of indirect pest control. In recent years, silence-based RNA interference (RNAi) has become an effective analytical tool for analyzing protein-specific functions [23,24,25,26,27]. Odor-binding proteins have a variety of functions, such as specifically recognizing and binding odor molecules, transporting odor molecules, and assisting in pheromone release, which is a key step in the process of insect olfactory recognition. Therefore, the binding of OBPs and odor molecules is a research hotspot in this field. At present, the role of OBPs in the olfactory mechanism of insects is mainly studied through fluorescence competitive binding, RNAi, EAG experiments, molecular docking, and other technologies [27,28,29,30,31]. The results show that the fluorescence competitive binding experiment and molecular docking technique are important methods to study the role of odor binding proteins in the olfactory mechanism of insects.

Regarding odor recognition of *G. qinghaiensis*, our group screened and identified 11 chemoreceptor proteins (CSP), 17 odor-binding proteins, and 12 ionoreceptors (IR) based on transcriptomic data of 4th instar larvae and male and female adults, and conducted bioinformatics analysis and expression pattern determination of these proteins [32,33,34,35,36]. At present, there are few studies on the genes of odor-binding proteins in *G. qinghaiensis*, and no reports on their functions have been reported. In this study, *GqinOBP10*, a classic OBP family with broad-spectrum expression in each tissue, was selected as the odor-binding protein in this study, and the function of *GqinOBP10* in the recognition of volatiles of the host plant (*F. rubra*) was further explored through prokaryotic expression, fluorescent competitive binding, molecular docking technology, and RNAi. This study provided a theoretical basis for exploring the role of *GqinOBP10* in the olfactory sensory mechanism of *G. qinghaiensis* and a new idea for the subsequent development of green control targets for this pest.

## 2. Results

### 2.1. Construction of Prokaryotic Expression Vector

**Figure 1:**
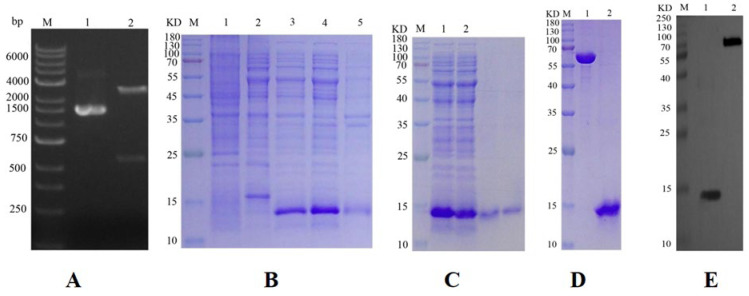
Prokaryotic expression and purification. (**A**): Electrophoretic identification of cloned plasmid pMD 19-T-GqinOBP10 by double enzyme digestion, Lane 1: Plasmid; Lane 2: Plasmid Digested with Mlul-Xbal; Lane M: DNA Marker. (**B**): GqinOBP10 protein expression identification results of SDS-PAGA analysis, M: Protein molecular quality standard; 1: pCZN1 induction (no load); 2: not induced; 3: After induction; 4: Supernatant after induced crushing; 5: Precipitate after induced crushing. (**C**): Results of SDS-PAGA analysis of GqinOBP10 protein purification, M: Protein molecular quality standard; 1: Processing samples after crushing; 2: outflow. (**D**): Results of SDS-PAGA detection of GqinOBP10 protein concentration, M: Protein molecular quality standard; 1: 0.5 mg/mL BSA; 2: Purified sample. (**E**): Western blot analysis of GqinOBP10 protein, M: Protein molecular quality standard; 1: purified sample; 2: GST/His positive.

The results of the double enzyme in Figure 1A. The plasmid in lane 1 displayed clear bands at approximately 1500 bp, while the digested product in lane 2 showed bands at 500 bp, with the cut vector observable at 4000 bp. These results indicate that the enzyme digestion was successful and aligned with the expected outcomes.

### 2.2. Expression and Purification of Target Protein

The electrophoresis results are presented in Figure 1B. Both the product from lane 3 (induced) and the supernatant from lane 4 (cell lysate) exhibited target bands near 15 kDa. In contrast, no specific bands were detected in the empty expression vector or in the bacterial solution under non-inductive conditions, confirming that the extracted protein corresponds to the GqinOBP10 target protein, which was effectively expressed following induction. The electrophoretic results indicated that OBP10 was present in both the supernatant and the pellet; however, the expression level in the supernatant was significantly higher than that in the precipitate. The size of the specific protein band and the length of the His tag sequence were consistent with the predicted molecular weight of the protein.

GqinOBP10 was purified using Ni column affinity chromatography, and the purity of the protein was verified by SDS-PAGE, as shown in Figure 1C. The results indicated that the purified protein solution produced a distinct single band near 15 kDa, confirming that the purification of GqinOBP10 was successful and met quality expectations. Subsequent to the removal of the His tag by recombinant enterokinase, SDS-PAGE was performed to evaluate the quality of the purified GqinOBP10 solution in comparison with a 0.5 mg/mL standard protein solution. The results, displayed in Figure 1D, demonstrated that the GqinOBP10 solution, post-tag removal, still exhibited clear bands at 15 kDa, meeting the expected standards and confirming its suitability for subsequent fluorescence competitive experiments.

### 2.3. Target Protein Quality Analysis

The purified protein sample demonstrated effective antigenicity when combined with rabbit anti-rat and sheep anti-rabbit antibodies at a dilution ratio of 1:10,000, resulting in the observation of distinct bands. These findings indicate that the prepared protein possesses high quality and sensitivity. Quality control (QC) testing revealed that the protein concentration was 0.6 mg/mL, with a purity exceeding 85%. The protein product was stored in PBS buffer and preserved at −80 °C for future applications (Figure 1E).

### 2.4. Analysis of Fluorescence Competitive Binding Characteristics of GqinOBP10 and Odor Ligands

**Figure 2:**
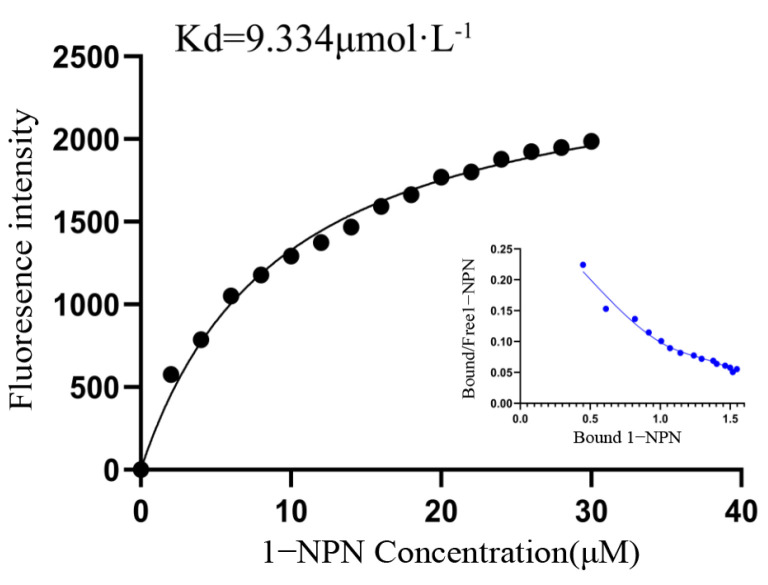
Binding of GqinOBP10 to 1-NPN fluorescent probe.

In this experiment, the His tag was removed from GqinOBP10 to prevent any adverse effects on the solubility and functional structure of the odor-binding protein. The binding curve for GqinOBP10 and the 1-NPN fluorescence probe is illustrated in Figure 2. The maximum fluorescence intensity was observed at 407 nm. As the concentration of 1-NPN increased, the maximum fluorescence value of the complex formed between 1-NPN and GqinOBP10 gradually increased, eventually reaching a saturation point, indicating strong binding characteristics between GqinOBP10 and 1-NPN.

This analysis suggests that the binding interaction between 1-NPN and GqinOBP10 occurs in a 1:1 stoichiometry. Therefore, 1-NPN can be utilized as a fluorescence probe for subsequent fluorescence competitive binding assays.

In this study, the binding characteristics of GqinOBP10 were evaluated through a fluorescence competitive binding assay using eight odor molecules as ligands. The dissociation constants Ki of eight ligand substances, 1-Aminocyclopropane carboxylic acid, 1-indenol, 1, 4-cyclohexanedione, 2-Amino-1-phenylethanol, β-cyano-L-alanine, 2-Oleoylglycerol, 3-Aminoisobutanoic acid, 1,2, 4-butantriol, were calculated by Scatchard equation. The obtained values were 18.59, 11.94, 13.04, 4.48, 12.21, 5.96, 14.88, and 11.58 μmol·L^−1^, respectively. These results indicate that GqinOBP10 exhibits a strong binding affinity for the volatiles of the eight plant volatiles (with IC_50_ < 40 μmol·L^−1^ and Ki < 20 μmol·L^−1^) (Table 1).

**Figure 3:**
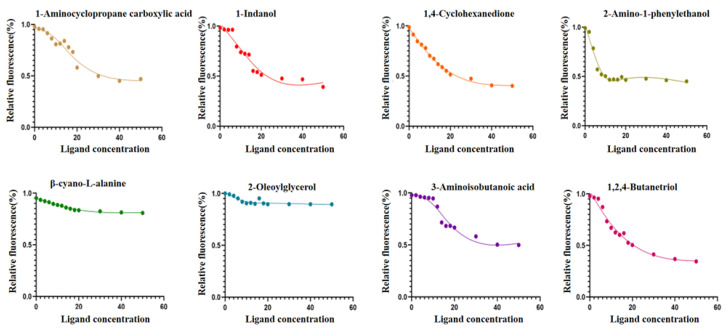
Competitive binding of ligands to 1-NPN fluorescent probes.

GqinOBP10 demonstrated a broad-spectrum binding affinity, with the strongest interaction observed with 2-amino-1-phenylethanol (Ki = 4.48 μmol·L^−1^), suggesting that the protein has favorable binding properties with alcohols. The competitive binding curve of GqinOBP10 with the eight ligands and the fluorescent probe 1-NPN is depicted in Figure 3. Using 2-amino-1-phenylethanol as a reference, all ligands were able to successfully displace the fluorescent probe from the GqinOBP10-probe complex; however, their binding capabilities varied. Notably, 1,2,4-butanetriol exhibited the strongest affinity, resulting in a reduction in fluorescence intensity to less than 35%.

### 2.5. Construction and Evaluation of OBP10 Protein 3D Model

**Figure 4:**
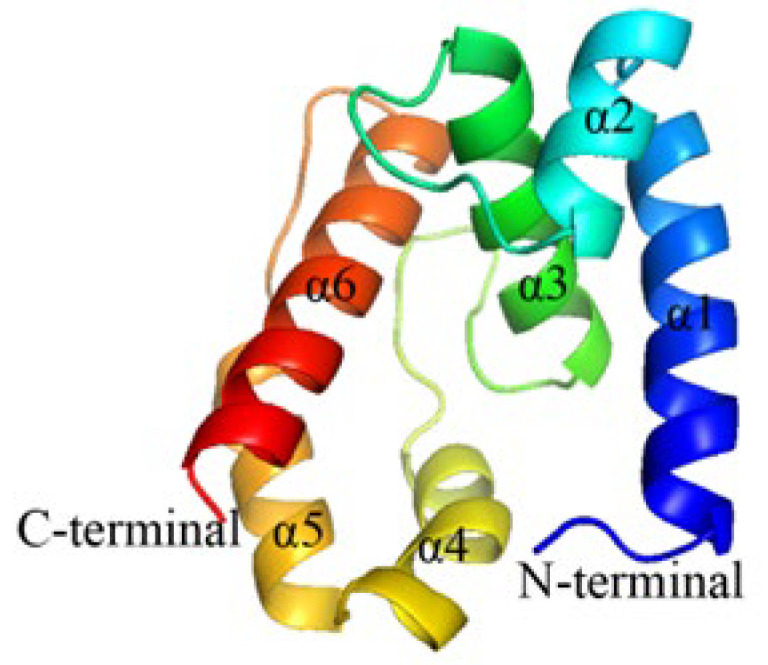
Tertiary structure of OBP10 in *G. qinghaiensis*.

**Figure 5:**
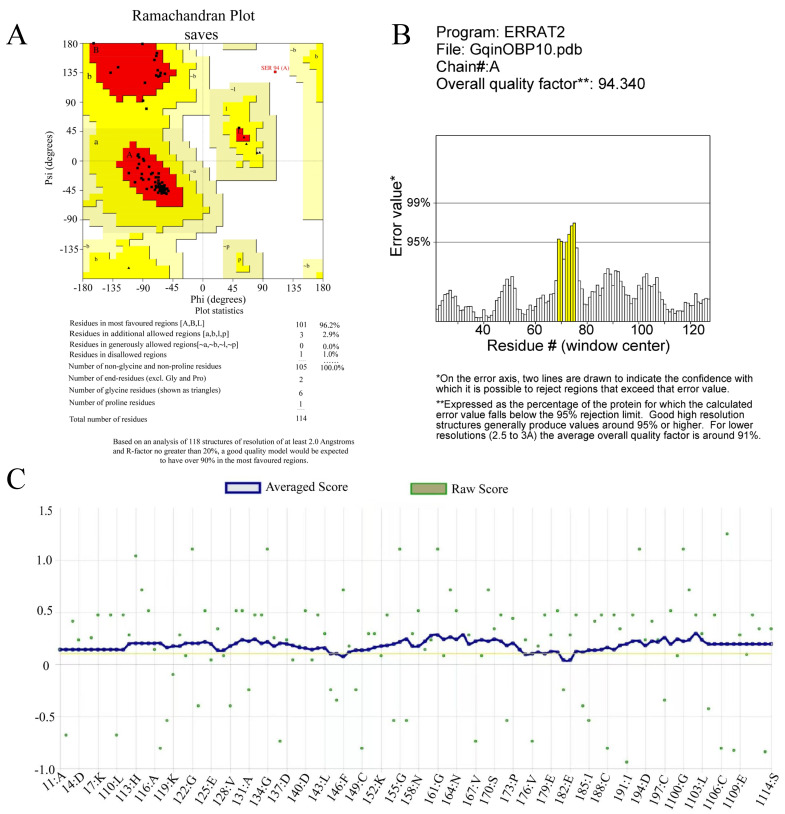
Ramachandran plot, ERRAT and Verify 3D evaluation result for OBP10 3D model evaluation. (**A**): Ramachandran plots of GqinOBP10 protein model. (**B**): Error values of GqinOBP10 model evaluated by ERRAT. (**C**): Verify-3D scores for GqinOBP10 protein model.

The GenBank entry number for the OBP10 gene sequence of *G*. *qinghaiensis* is ON365530, with a sequence length of 405 bp and an amino acid length of 134 residues. The results of the tertiary structure modeling for OBP10 are illustrated in Figure 4. The 96.2% of the model resides in the favored red region, with an additional 2.9% located in the yellow region, indicating allowable conformations. This suggests that the protein structure model is reliable (Figure 4). Furthermore, Analyze the number of non-covalent bonds formed between different atomic types within a 0.35 nm range of the protein. The Verify-3D program was utilized to evaluate the model’s rationality, yielding an ERRAT score of 94.3396. Additionally, 96.49% of the amino acid residues were found to fall within the reasonable range according to Verify-3D analysis (Figure 5). Overall, the results of these evaluations indicate that the OBP10 protein model is both reasonable and reliable.

### 2.6. Molecular Docking of GqinOBP10 with Ligands

**Figure 6:**
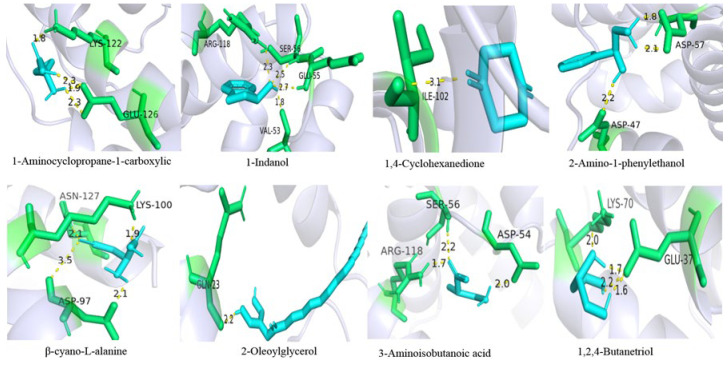
The binding of GqinOBP10 to 8 odor molecules.

The binding energies of GqinOBP10 when docked with various organic molecules were all negative (Table 2). The binding energies for 1-Aminocyclopropane carboxylic acid, 1-Indanol, 1,4-Cyclohexanedione, 2-Amino-1-phenylethanol, β-cyano-L-alanine, 2-Oleoylglycerol, 3-aminoisobutyric acid, and 1,2,4-butanetriol were measured at −3.48 kJ/mol, −4.79 kJ/mol, −4.04 kJ/mol, −4.36 kJ/mol, −3.73 kJ/mol, −6.92 kJ/mol, −3.08 kJ/mol, and −1.34 kJ/mol, respectively. The van der Waals forces for these eight odor molecules were −2.11 kJ/mol, −4.92 kJ/mol, −3.86 kJ/mol, −3.37 kJ/mol, −2.73 kJ/mol, −10.16 kJ/mol, −3.05 kJ/mol, and −2.42 kJ/mol, respectively. Molecular docking revealed that OBP10 and all ligands interacted at 1–4 amino acid binding sites. Specifically, the docking results for GqinOBP10 with three volatile odor compounds—2-Amino-1-phenylethanol, β-cyano-L-alanine, and 1,2,4-butanetriol—indicated that these three ligands could bind to the same amino acid sites. Notably, three residues, Asp57, Asp97, and Glu37, formed multiple hydrogen bonds, enhancing the stability of the protein-ligand interactions (Figure 6).

### 2.7. Effect of RNA Interference on the Expression Level of GqinOBP10

**Figure 7:**
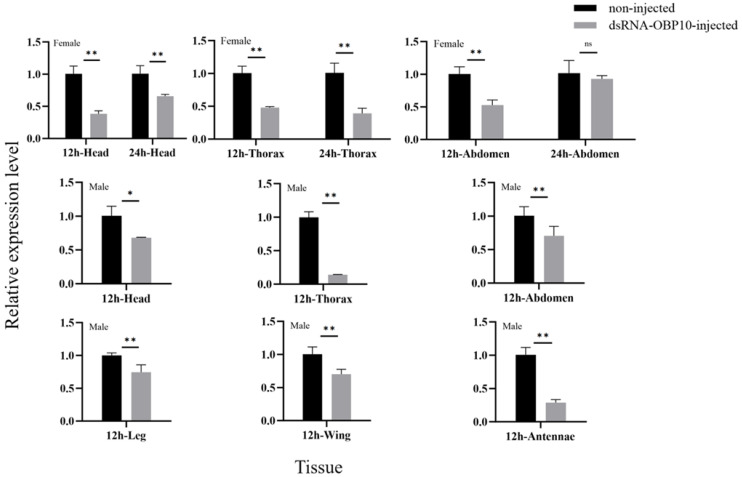
Relative expression levels of the injected *dsOBP10* gene versus those without *dsOBP10* injection. ns: not significant; * and ** indicate that the relative expression levels are significantly different at the 0.05 and 0.01 levels (*t*-test).

Our results demonstrated that *dsOBP10* injection significantly reduced the expression of the target gene in both male and female tissues at both 12 h and 24 h post-injection (*p* < 0.05) compared to the control group (Figure 7). In female tissues, there was a notable decrease in expression levels in the head, thorax, and abdomen after 12 h of interference. After 24 h, the expression level in the control group remained stable, while the expression in the experimental group either remained unchanged or increased. This observation may indicate a decrease in the activity of the interfering reagent over time.

In male tissues, expression levels also decreased after 12 h of interference, with particularly significant reductions observed in the antennae (approximately threefold decrease) and thorax (27-fold decrease) compared to the control group. These findings suggest that *dsRNA* injection is an effective method for reducing the expression levels of *GqinOBP10* mRNA, thereby allowing us to proceed with further investigations into antennal function and potential detection.

### 2.8. EAG Response After RNA Interference

**Figure 8:**
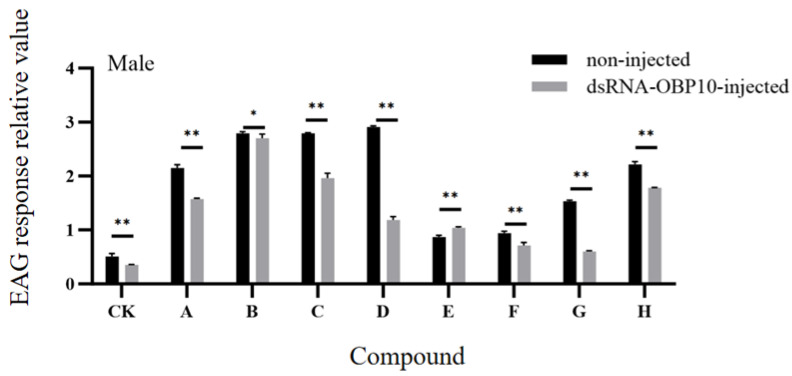
EAG response levels of *G. qinghaiensis* to each compound with and without *dsOBP10* injection. A–H: 1,4-Cyclohexanedione, 1,2,4-butanetriol, 3-Aminoisobutanoic acid, 1-Indanol, β-cyano-L-alanine, 2-Amino-1-phenylethanol, 2-Oleoylglycerol, 1-Aminocyclopropane-1-carboxylic acid. * and ** indicate that the relative expression levels are significantly different at the 0.05 and 0.01 levels (*t*-test).

Following the injection of dsOBP10 into male adults, we assessed the electroantennographic (EAG) responses of their antennae to eight plant volatiles in both the experimental and control groups. Compared to the solvent control (n-hexane), the antennae exhibited significantly higher EAG responses to all eight compounds, with varying levels of sensitivity to different stimuli. Among these, the strongest responses in the male control group were elicited by 1,4-cyclohexanedione (A), 1,2,4-butanetriol (B), 3-aminoisobutanoic acid (C), 1-indanol (D), and 1-aminocyclopropane carboxylic acid (H) (Figure 8).

However, after silencing the OBP10 gene, we observed a certain degree of reduction in the EAG responses to the seven tested compounds. Notably, the antennal potential values for 1-indanol (D) and 2-Oleoylglycerol (G) decreased by more than two to three times compared to the control group. Interestingly, treatment with dsOBP10 resulted in a stronger EAG response to β-cyano-L-alanine (E), suggesting that multiple odor-binding proteins may facilitate the recognition of this particular compound.

## 3. Discussion

*G. qinghaiensis* is a significant pest in the alpine meadows of the western plateau of China. Its biological behavior is influenced by a complex synergy of various environmental chemicals. Odorant-binding proteins (OBPs) play a crucial role in this context by dissolving and transporting odor molecules, such as plant volatiles, through lymphatic fluid, thereby facilitating the olfactory sensing mechanism of these caterpillars [34]. By identifying key *OBP* genes in insects, we can study their corresponding target ligands more effectively so as to reduce the use of chemical pesticides, minimize environmental pollution, and achieve sustainable pest control. Lepidopteran OBPs can be classified into several categories, including pheromone-binding proteins (PBPs), general odorant-binding proteins (GOBPs), and the antennal-binding protein X (ABPx) [37]. Furthermore, based on the number and spacing of cysteines in their amino acid sequences, OBPs can be categorized into five types: Classic OBPs, Dimer OBPs, Plus-C OBPs, Minus-C OBPs, and Atypical OBPs [38]. In our previous experiments, we established that GqinOBP10 belongs to the Classic OBP category; however, the specific function of GqinOBP10 in the interaction mechanism between grassland caterpillars and host plant volatiles remains unknown. This study aims to elucidate this function by constructing an expression vector and analyzing the characteristics of fluorescence competitive binding between purified GqinOBP10 and eight ligands. Additionally, we performed homology modeling and molecular docking to simulate and verify the binding interactions of GqinOBP10 with these ligands. Ultimately, this research seeks to explore the specific role of GqinOBP10 in the olfactory mechanism that enables grassland caterpillars to recognize host plant volatiles, thereby providing a theoretical foundation for further studies on the olfactory mechanisms of these pests. This work also offers a novel approach to the green control of major grassland pests in China.

To enhance the likelihood of obtaining soluble proteins, the signal peptide sequence was removed, and the His tag was also eliminated from GqinOBP10. These modifications aimed to preserve the solubility and functional structure of the odor-binding protein. After cell disruption and subsequent isolation of components, a significant number of proteins were detected in the supernatant, while some proteins were found in the precipitate. This observation may be attributed to incomplete separation or the influence of disulfide bonds within OBP10.

Odor-binding proteins are the first biochemical reaction in insects to recognize external odor volatiles. In the interaction mechanism between insects and plants, the host plant volatiles guide insects in locating food sources, oviposition sites, and mates, as well as in gathering information about natural enemies [39]. Therefore, examining the relationship between OBPs and odor volatiles can shed light on their binding mechanisms. Additionally, it can facilitate the design of active volatiles that influence insect behavior by leveraging the structural features of OBPs. In our previous experiment, we investigated the expression of *GqinOBP10* in male and female adults as well as in larvae. We found that the expression levels of *GqinOBP10* were significantly higher in male and female caterpillars compared to females alone. Additionally, there was notable expression in young larvae (3–4 instars) during the gluttonous stage, when they consume large amounts of forage grass [34,35]. These findings suggest that the *GqinOBP10* gene plays a crucial role in larvae’s odor perception of host plant volatiles and serves different functional roles in olfactory perception and mating behaviors in both sexes. This results in distinct expression patterns and behavioral responses. However, further experimental validation is needed to confirm this hypothesis. The fluorescence competitive binding assay serves as an effective method for screening proteins, identifying specifically bound ligands, and analyzing their interactions. This technique provides a visual representation of the binding affinity of OBPs to ligands by measuring changes in fluorescence intensity [40,41,42]. Given that *G. qinghaiensis* are significant pests in alpine meadows and primarily feed on high-quality grasses within the grass family, the insects examined in this study were collected from pastures in Haibei Prefecture, Qinghai Province. The primary food source for these collected caterpillars was identified as *Festuca rubra*. Consequently, eight odorant compounds with abundant content from *F. rubra*, the host plant on which the caterpillars feed, were chosen as ligands for the fluorescence competition assays. The study revealed that GqinOBP10 exhibited a robust binding affinity to the odor volatiles emitted by host plants. Notably, it showed the highest binding affinity to 2-Amino-1-phenylethanol, and also demonstrated significant binding to the other seven ligands tested. These findings suggest that *OBP10* plays a crucial role in the host plant localization process of *G. qinghaiensis*. This discovery provides a solid foundation for future investigations into the functional role of *OBP10* in these pests.

Through homology modeling, we determined that GqinOBP1 possesses six α-helix structures, characteristic of odor-binding proteins (OBPs). Molecular docking technology allows for the virtual calculation of the binding affinity between receptor proteins and ligand molecules, providing a visual representation of the binding mode. This approach serves as a valuable reference for studying protein function and the molecular recognition mechanisms involved [43]. In this study, the binding energy of the eight ligands was negative, and GqinOBP10 could bind to eight odorant volatiles, including alcohols, ketones, esters, and carboxylic acids, which was consistent with the results that OBPs could bind a variety of odor substances with different chemical groups. There are differences in binding capacity, which may be due to the nature of the chemical itself affecting the binding ability of OBPs. Some studies have shown that within a certain range, the increase in the number of carbon atoms in odor volatiles will lead to the weakening of the binding ability of OBPs to ligand molecules [31,44], and some studies have concluded that the strength of protein binding ability is not significantly related to the number of ligand carbon atoms [45]. In this experiment, the protein has a strong binding ability to a ligand with a large number of carbon atoms, which may be related to the large space occupied by the conformation of the ligand formation mode with a large number of carbon atoms [46]. In addition, it has been shown that the shape of the ligand also affects the binding ability of OBPs, and the binding ability of OBPs to cyclic ligands is stronger than that of chain ligands [47]. The effective binding of OBPs to ligand species is dependent on the interaction force of amino acids and ligand formation of proteins. The main way for GqinOBP10 to bind to ligands is through hydrophobic, van der Waals forces and hydrogen bonding, which conforms to the characteristics of OBPs, a transporter; that is, through the change in protein conformation, it binds and releases ligand molecules, so it mostly binds to ligand molecules with non-covalent interaction forces, which is convenient for the release of subsequent ligand molecules. This is similar to the binding patterns of *Plutella xylostella* PxylOBP33 [46], *Agrilus zanthoxylumi* AzanOBP3 [47], and *Dastarcus helophoroides* DhelOBP21 [48]. Furthermore, this study revealed that the amino acid aspartic acid (Asp) appeared five times during the binding of GqinOBP10 to the compounds. This suggests that Asp may play a crucial role in the recognition of these compounds by the protein [49].

RNA interference (RNAi) technology has been employed to study gene function in insects through the injection of double-stranded RNA (dsRNA) [50]. By targeting insect *OBP* genes, this technique can disrupt olfactory function, alter electrophysiological responses, modify odor preferences, and influence insect growth and development [51,52]. In this study, we examined the tissue expression levels and electroantennogram (EAG) response levels of *dsOBP10* in both the control and experimental groups following interference with *OBP10*. The results indicated that, compared to the control group, the expression levels of the target gene in male and female tissues were significantly reduced 12 and 48 h after the injection of *dsOBP10* (*p* < 0.05). In female tissues, after 24 h, the expression level in the control group remained relatively stable, while the expression level in the experimental group either remained unchanged or increased. This change may be attributed to a decrease in the efficacy of the interfering reagent over time. In male adults, 12 h post-injection of *dsOBP10*, the EAG response levels to seven compounds were significantly reduced (*p* < 0.05), particularly for 1-Indanol and 2-Oleoylglycerol. Molecular docking experiments further confirmed that the binding affinity between OBP10 and 2-Oleoylglycerol was the strongest among the eight tested substances. Thus, silencing *GqinOBP10* significantly impairs the olfactory perception of host volatiles in *G. qinghaiensis*, providing a foundation for the development of effective attractants.

## 4. Materials and Methods

### 4.1. Insects

Larvae were collected in June 2023 from Haibei Tibetan Autonomous Prefecture, Haiyan County, Qinghai Province (36°59′06.6″ N, 100°52′19.1″ E, altitude 3095.1 m). They were reared in the laboratory until male and female adults emerged. The antennae were then dissected and immediately frozen in liquid nitrogen before being stored at −80 °C for later use.

### 4.2. Cloning, Prokaryotic Expression, and Purification of OBP10 Gene

Collected Fifty antennae of *Gynaephora qinghaiensis*. Total RNA was extracted from the antennae using the TaKaRa (Baosheng Bioengineering Co., Ltd., Beijing, China) total RNA kit to synthesize the first strand of cDNA. Based on the OBP10 gene sequence (GenBank accession number: ON365530) [32], primers were designed with the signal peptide sequence excluded. The cDNA was amplified via PCR, and the resulting product was ligated into the pMD19-T vector. The successfully ligated vector was then transformed into DH5α competent cells, from which plasmids were extracted. The extracted cloned plasmid pMD19-T and the expression vector pCZN1 were subjected to double digestion using the restriction endonucleases MluI and XbaI. The resulting DNA fragments were ligated to the expression vector pCZN1 using T4 ligase at 16 °C overnight. The expression vector containing the target gene was transformed into BL21 (DE3) cells. Cellular OD600 is measured at induction. 

During the transformation process, isopropyl β-D-1-thiogalactopyranoside (IPTG) as an inducer was added to the medium when plating (final concentration of 1 mM), and the bacteria were finally shaken and cultured. Afterward, the bacterial culture was harvested by centrifugation, and cell lysis was performed to separate the supernatant and precipitate. Protein expression was assessed using SDS-PAGE. The His-tagged OBP10 was purified via Ni column affinity chromatography. After purification, the His tag was removed using recombinant enterokinase to obtain the target protein GqinOBP10. The concentration of GqinOBP10 was determined using a BCA protein concentration assay kit, and the protein was stored at −20 °C for future use.

### 4.3. Western Blot Analysis of Purified GqinOBP10

Rabbit anti-rat and sheep anti-rabbit antibodies labeled with horseradish peroxidase (HRP) were prepared. A polyacrylamide gel of the appropriate concentration was cast, and the electrophoresis apparatus was set up according to standard SDS-PAGE procedures. Following electrophoresis, the gel was soaked in transfer buffer for 5–10 min. A PVDF membrane was activated by soaking in methanol, followed by rinsing with ultra-pure water until it became translucent. The transfer membrane was then immersed in transfer buffer prior to the transfer process. After the electric transfer was completed, the membrane was removed and washed with PBST (phosphate-buffered saline with Tween 20). It was subsequently placed on a shaking platform for 1 h. The primary antibody was diluted in PBST, while the secondary antibody was diluted in 5% milk. The membranes were incubated with both the primary and secondary antibodies sequentially (the enzyme-labeled secondary antibodies were HRP-labeled rabbit anti-mouse and sheep anti-rabbit). Finally, detection was performed using the enhanced chemiluminescence (ECL) method.

### 4.4. Determination of Fluorescence Competitive Binding Characteristics of GqinOBP10

The instrument utilized in this experiment was the 960PC fluorescence spectrophotometer (Shanghai Yidang Analytical Instrument Co., Ltd., Shanghai, China). The fluorescent probe, 1-NPN, was dissolved in HPLC-grade methanol to prepare a storage solution with a concentration of 1 mM. Eight ligands derived from host plant volatiles of purple fescue (*F. rubra*) were selected to compete with the fluorescent probe. These ligands included 1-aminocyclopropane carboxylic acid, 1-Indenol, 1,4-Cyclohexanedione, 2-Amino-1-phenylethanol, β-cyano-L-alanine, 2-Oleoylglycerol, 3-Aminoisobutyric acid, and 1,2,4-Butanetriol (detailed information regarding the compounds is presented in Table 1). Each ligand was dissolved in HPLC-grade methanol to achieve a final concentration of 1 mM for use in the binding assays. The purified protein solution, obtained through dialysis and ultrafiltration, was diluted with Tris-HCl buffer (50 mM, pH 7.4) to a final concentration of 2 µM for the binding assays.

### 4.5. Determination of Binding Constant of GqinOBP10 and 1-NPN

The excitation wavelength for 1-NPN was determined to be 370 nm, and the fluorescence intensity was measured within the wavelength range of 380 to 550 nm. To assess the binding interaction, 1-NPN solution was added dropwise to a quartz cuvette containing 1 mL of protein solution, with concentration increments of 2 µM each time. This procedure established a concentration gradient of 0, 2, 4, 6, 8, 10, 12, 14, 16, 18, and 20 µM. Measurements were taken until the fluorescence intensity reached a plateau, indicating that the concentration at this point represented the maximum binding affinity between GqinOBP10 and 1-NPN.The collected fluorescence data were analyzed using GraphPad Prism 9.5 software, where the Scatchard equation was applied in a nonlinear regression analysis to determine the binding constant (K1-NPN) of the protein to 1-NPN.

### 4.6. Determination of Binding Ability of GqinOBP10 to Ligand Substances

Initially, 1 mL of a 2 µM protein solution was placed in a cuvette, and 1-NPN was gradually added until the concentration reached the maximum binding value. The fluorescence intensity was measured after thorough mixing. Subsequently, the fluorescence competitive binding assays were conducted using the eight ligand solutions. The final concentrations of the ligands in the cuvette were varied to create a concentration gradient of 0, 2, 4, 6, 8, 10, 12, 14, 16, 18, 20, 25, and 30 µM. As the ligand concentration increased, a corresponding decrease in fluorescence intensity was observed, indicating that the fluorescent probe was being displaced from the protein by the ligands. The addition of ligands continued until the fluorescence intensity stabilized and no longer changed.

### 4.7. Homology Modeling and Molecular Docking

Homology modeling was performed using SWISS-MODEL (expasy.org) to obtain the three-dimensional structure of the protein. The quality of the generated protein model was assessed using the ERRAT program, as well as the Verify-3D and ProCheck programs developed by UCLA.

Molecular docking was conducted using AutoDock 4.2.6 software. Organic compounds were retrieved from PubChem, and their three-dimensional structures were downloaded. The downloaded compound information was then converted into PDB format using Open Babel GUI 3.1.1 software. The data models of GqinOBP10 and the eight odor molecules were prepared by dehydrating and treating them with AutoDock 4.2.6 to simulate the binding interactions between GqinOBP10 and the ligands.

### 4.8. RNA Interference

PGEM-T Easy Vector (Promega, Madison, WI, USA), Plasmid Extraction Kit (TIANGEN, Beijing, China), T7 RiboMAX™ Express RNAi System (Promega), Purification Kit (Promega), 3M NANC (Solarbio, Beijing, China). Based on the identified nucleotide sequence of the OBP10 gene in *G. qinghaiensis*, the following RNA interference primers were designed: Forward Primer (F): GTAATACGACTCACTATAGGGAGAGATTACCACCGCATGATTG, Reverse Primer (R): GTAATACGACTCACTATAGGGAGATAGCCATAGTAGTGACTCCAACG. Using male wing cDNA with high expression of *OBP10* as a template [34], the gene sequence containing the T7 promoter was amplified by RT-PCR. The amplification product was purified and then ligated into the PGEM-T Easy vector. The reaction mixture was incubated overnight at 16 °C for ligation. The ligation product was transformed into Escherichia coli competent cells (DH5α). After transformation, samples were sent for sequencing, and the correct bacterial colonies were cultured for plasmid extraction. The obtained correct plasmid served as a template for the large-scale synthesis of double-stranded RNA (dsRNA). dsRNA was synthesized using the T7 RiboMAX™ Express RNAi System (Promega), with the purified PCR amplification product as the template.

Using a microsyringe, 1 μL (1000 ng/μL) of *dsRNA* was injected into the internode membrane between the abdominal ends of 1-day-old male and female moths. The control group received an equivalent volume of ddH_2_O. Care was taken to keep the syringe needle as parallel to the abdominal epidermis as possible during the injection to ensure accuracy and minimize harm. Each treatment consisted of three biological replicates, with each replicate containing 30 male and female adults.

To investigate the interference efficiency of dsOBP10 in *G. qinghaiensis*, we measured the expression levels of dsOBP10 using quantitative reverse transcription PCR (qRT-PCR) at 12 and 24 h post-injection. Samples were collected after 12 h (for male and female adults) and 48 h (for female adults) post-injection. Due to the short lifespan of male adults, which typically resulted in death within one day of injection, the time points were chosen accordingly. Each male and female adult caterpillar was injected with 1 μL of either ddH_2_O or dsOBP10. RNA was then extracted from various tissues of female adults (head, thorax, abdomen, legs, wings, and antennae) to assess the expression levels of the target gene. Each experiment was conducted with three biological replicates and three technical replicates to ensure the accuracy and reliability of the results.

To assess the antennal potential in male adults following different injection treatments, we selected eight volatiles from the plant volatiles of the moths. The antennae of the male adults were gently plucked from the root of the head using forceps, excising them at both the base and tip. The cut antennae were then attached between electrodes using Spectra 360 electrode gel (Parker Laboratories, Fairfield, NJ, USA), ensuring that the air outlet faced the antennae. Eight compounds selected for electroantennography (EAG) measurements were uniformly diluted to a concentration of 10 μg/μL using n-hexane as the solvent, with n-hexane employed as the negative control. Each volatile component was placed into a Pasteur tube and connected to an air stimulation controller. EAG responses were recorded for 5 s, with stimulation intervals set at 15 s, and both stimulation and airflow maintained at a rate of 4 mL/s. Each compound was tested three times, with six antennae (6 moths) assessed for each host plant volatile component. Blank control measurements of the antennal potential were taken at the beginning and end of each experimental set. A total of 200 insect specimens were used for the EAG reactions.

### 4.9. Data Analysis

Statistical analysis of the relative expression levels and antennal potential values of genes in different tissues after RNA interference was conducted using SPSS Statistics 26.0. A *T*-test (*p* < 0.05) was performed to determine statistical significance. The tissue expression profiles and differences in antennal potential were visualized using GraphPad Prism 9.5 software. Use GraphPad Prism 9.5 to perform One-Way Analysis of Variance (One-Way ANOVA) on the EAG response data of different compounds (*p* < 0.05) and plot the data. The reference values of EAG relative response refer to previous studies [53].

## Figures and Tables

**Table 1:** Basic information and binding characteristics of 8 odor molecules in plant volatiles.

Ligand	CAS NO.	Purity	Chemical Construction	Molecular Formula	IC_50_ (μmol·L^−1^)	K_i_ (μmol·L^−1^)
1-Aminocyclopropane-1-carboxylic acid	22059-21-8	≥98%	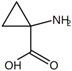	C_4_H_7_NO_2_	18.7	18.59
1-Indanol	6351-10-6	≥98%	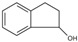	C_9_H_10_O	12.01	11.94
1,4-Cyclohexanedione	637-88-7	≥98%	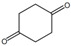	C_6_H_8_O_2_	13.12	13.04
2-Amino-1-phenylethanol	7568-93-6	≥98%	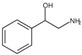	C_6_H_8_O_2_	4.506	4.48
β-cyano-L-alanine	6232-19-5	≥98%	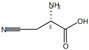	C_4_H_6_N_2_O_2_	12.29	12.21
2-Oleoylglycerol	3443-84-3	≥98%	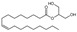	C_21_H_40_O_4_	5.997	5.96
3-Aminoisobutanoic acid	144-90-1	≥98%	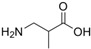	C_4_H_9_NO_2_	14.97	14.88
1,2,4-butanetriol	3068-00-6	≥98%	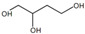	C_4_H_9_NO_2_	11.65	11.59

**Table 2:** Molecular docking result of 8 odor molecules in plant volatiles.

Classification	Ligand	Binding Energy	Hydrophobic Interaction	Van der Waals Force	Hydrogen Bond
Carboxylic acid	1-Aminocyclopropane-1-carboxylic acid	−3.48	Lys122	−2.11	Lys122; Glu126
Alcohol	1-Indanol	−4.79	Arg118; Tyr121; Lys122	−4.92	Val53; Glu55; Ser56; Arg118
Ketone	1,4-Cyclohexanedione	−4.04	Phe80; Thr85; Val101; Ile102; Tyr124	−3.86	Ile102
Alcohol	2-Amino-1-phenylethanol	−4.36	Gln52	−3.37	Asp47; Asp57; Asp57
Carboxylic acid	β-cyano-L-alanine	−3.73	Asp97; Asn127	−2.73	Asp97; Asp97; Lys100; Asn127
Ester	2-Oleoylglycerol	−6.92	Leu27; Leu68; Ile71; Phe80; Thr85; Tyr121	−10.16	Gln23
Carboxylic acid	3-Aminoisobutanoic acid	−3.08	Asp54; Glu55; Ser56	−3.05	Asp54; Ser56; Arg118
Alcohol	1,2,4-butanetriol	−1.34	Lys69	−2.42	Glu37; Glu37; Lys70

## Data Availability

The original contributions presented in the study are included in the article; further inquiry can be directed to the corresponding author.
